# Plausible Role of Asthma Biological Modifiers in the Treatment of Eosinophilic Esophagitis

**DOI:** 10.7759/cureus.16460

**Published:** 2021-07-18

**Authors:** Timothy C Olsen, Robert A Promisloff, Denise DeCostanzo, Gang He, Anthony M Szema

**Affiliations:** 1 School of Medicine, University of Rochester School of Medicine and Dentistry, Rochester, USA; 2 Medicine, Drexel University College of Medicine, Philadelphia, USA; 3 Pathology, Huntington Hospital, Huntington, USA; 4 Pathology, North Shore Digestive Medicine, Smithtown, USA; 5 Allergy and Immunology, Donald and Barbara Zucker School of Medicine at Hofstra / Northwell, Hempstead, USA

**Keywords:** eosinophilic esophagitis, biological modifiers, benralizumab, t-helper cells, eosinophils, endoscopy, ppi, steroid, gastroesophageal reflux disease, asthma

## Abstract

Eosinophilic esophagitis (EoE) is a T-helper type-2 (Th2/T2) cell-mediated disease characterized by 15 or more eosinophils per high-powered esophageal biopsy microscopy field (eos/hpf), excluding other causes. EoE is often clinically characterized by symptoms such as dysphagia, nausea, food impaction, and chest pain that do not respond to antacids. Two-thirds of patients are unresponsive to proton pump inhibitors (PPIs). Steroids may be effective but pose long-term health risks and can lose efficacy in patients with serum eosinophilia greater than 1,500 cells/µL. Because EoE is not IgE-mediated, allergy skin testing for food may benefit a subset of patients. These therapies have shortcomings, which necessitate further investigation. Herein, we report a patient successfully treated with benralizumab (anti-IL-5Rα), demonstrating a potential solution to the lack of effective treatments for EoE.

## Introduction

First reported in 1977, eosinophilic esophagitis (EoE) describes the hyperinflammatory eosinophilic infiltration and narrowing of the esophagus, immunologically akin to asthma [1]. Current treatments comprise proton pump inhibitors (PPIs), diet modification, corticosteroids, and esophageal dilation. 

However, PPIs are ineffective for nearly two-thirds of EoE patients [2]; dietary modifications are difficult to maintain [2]; and corticosteroids (viscous budesonide) may increase the risk for hypothalamic-pituitary-adrenal axis suppression, oral candidiasis, and other side effects [3]. Moreover, among the 33% of EoE patients who receive endoscopic dilation, approximately 58% of them require additional dilations, potentiating the risk for fibrostenotic progression and perforation [4].

As of March 2021, current treatments are largely ineffective, and none are labeled for EoE [5-6]. However, benralizumab may serve as an effective treatment option, as demonstrated in our case’s reduction of esophageal eosinophils and symptoms.

## Case presentation

We administered a free sample dose of benralizumab (30 mg subcutaneous) in an off-label manner to treat a patient with EoE who did not tolerate steroids and PPI treatment. This patient preferred to avoid long-term use of steroids, with their attendant side effects.

A 27-year-old woman diagnosed with EoE by her gastroenterologist was referred to us after one year of failing to respond to optimal anti-reflux measures, including pantoprazole treatment and diet modification (caffeine restriction). Moreover, a six-food elimination diet did not suffice either. Her previous gastroenterologist also placed her on fluticasone/vilanterol 100 mcg-25 mcg/inh inhalation powder one puff swallowed once a day (without a spacer). Shortly thereafter, the patient refused her treatment due to concerns about the potential consequences of consuming a steroid suspension on her singing. Furthermore, she showed no improvement on her steroid solution regimen. 

**Figure 1:**
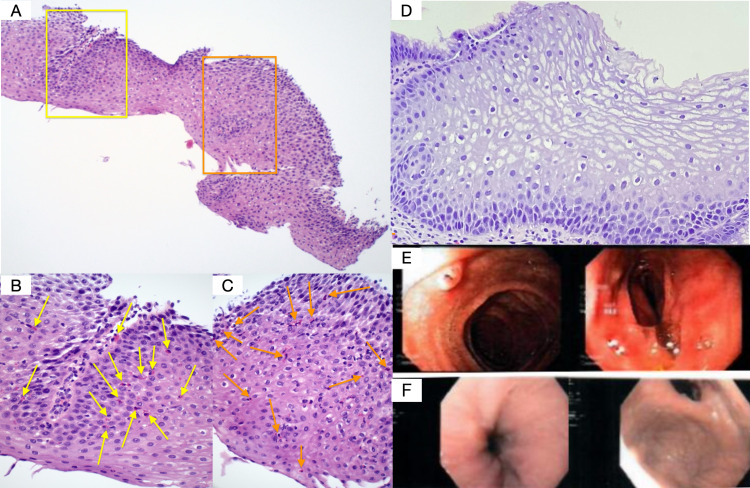
Esophageal pathology and endoscopy. Pathology slides of the patient’s esophagus pre (A-C) and post (D) benralizumab treatment. The yellow and orange squares (A) represent the field of views corresponding with the magnified pathology slides (B) and (C), respectively. The arrows (B-C) indicate eosinophils. Endoscopic images showcase the patient’s esophagus pre (E) and post (F) benralizumab treatment. More specifically, longitudinal furrows accompany esophageal inflammation and erythema pre-treatment (E) due to mucosal infiltration of eosinophils (A-C) which subsided following anti-IL-5 therapy, benralizumab (D)(F).

On her initial visit with us, the patient complained of minor dysphagia, spontaneous nausea, general discomfort in her throat, and difficulty swallowing pills. Her percutaneous skin-testing for 30 different food allergies tested negative; spirometry was normal. Her family history was unremarkable besides having a mother with multiple sclerosis and hypertension and a brother with allergies and asthma. Her blood analysis showed 400 absolute eosinophils. The patient’s endoscopy showed esophageal inflammation and erythema (Figure 1). Her initial esophageal biopsy revealed more than 15 eos/hpf, supporting the diagnosis of EoE. 

Five days after receiving benralizumab (upon the patient's consent), her symptoms completely subsided. One month later, her blood eosinophil count was zero. Esophageal mucosa appeared normal one month after benralizumab treatment. She was asymptomatic for 2.5 months following administration of benralizumab, but symptoms such as nausea slightly recurred post 2.5 months.

## Discussion

**Figure 2:**
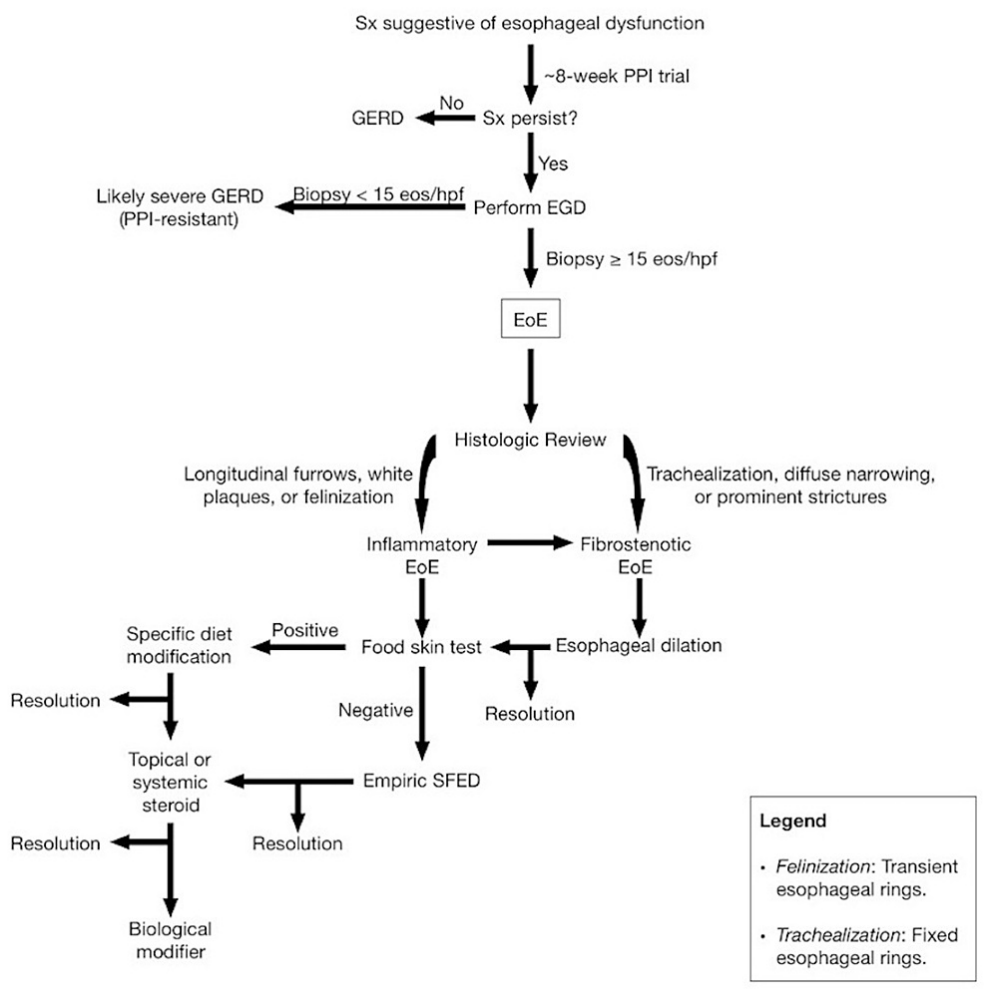
Modus operandi for assessment and treatment of EoE. Sx, symptoms; PPI, proton pump inhibitor; GERD, gastroesophageal reflux disease; EGD, esophagogastroduodenoscopy; eos, eosinophils; hpf, high powered field; EoE, eosinophilic esophagitis; SFED, six food elimination diet (wheat, milk, egg, nuts, soy, fish, and shellfish). *Note*: Original artwork created by Timothy Olsen BS, on September 17, 2020.

The phenotype for EoE typically presents as dysphagia, upper chest pain, nausea, and/or choking from food impaction [7]. The standard first line of management for symptoms suggestive of EoE involves approximately eight weeks of PPI treatment. If symptoms continue, then EoE is a likelier diagnosis, and an esophagogastroduodenoscopy (EGD) is required to differentiate EoE from gastroesophageal reflux disease (GERD). EoE confirmation entails detecting 15 eos/hpf or more in an esophageal biopsy, while seven or less is likely GERD (Figure 2) [4]. 

Currently, four Food and Drug Administration (FDA)-approved anti-eosinophil medications for asthma exist -- benralizumab, mepolizumab, reslizumab, and dupilumab. Our selection of Benralizumab was in part due to its relatively low percentage of reported adverse reactions. For example, during a 28-week clinical trial comparing benralizumab-treated asthma patients with placebo-treated asthma patients, the only statistically significant adverse reactions that occurred with either equal or more than 3% incidence comprised 8.2% experiencing headaches versus 5.3%, respectively, and 2.7% experiencing pyrexia versus 1.3%, respectively [8]. Moreover, benralizumab has a high specificity for lowering eosinophils [9]. It may thus bypass many of the traditional inefficiencies associated with current approaches by targeting the disease’s underlying immunopathogenesis more directly, as demonstrated in our patient’s treatment. 

According to theFDA, the treatment's primary contraindication is hypersensitivity to benralizumab or any of its excipients [8]. The recommended dose and administration of benralizumab is 30 mg administered subcutaneously once every four weeks for the first three doses, followed by once every eight weeks thereafter [8]. Benralizumab has an absorption half-life of approximately 3.6 days and an estimated absolute bioavailability of about 58% following subcutaneous administration either to the thigh, abdomen, or arm (no clinical difference in relative bioavailabilities) [8]. The medication is a humanized monoclonal antibody (IgG1/κ-class) sourced from Chinese hamster ovary cells with recombinant DNA technology [8].

**Figure 3:**
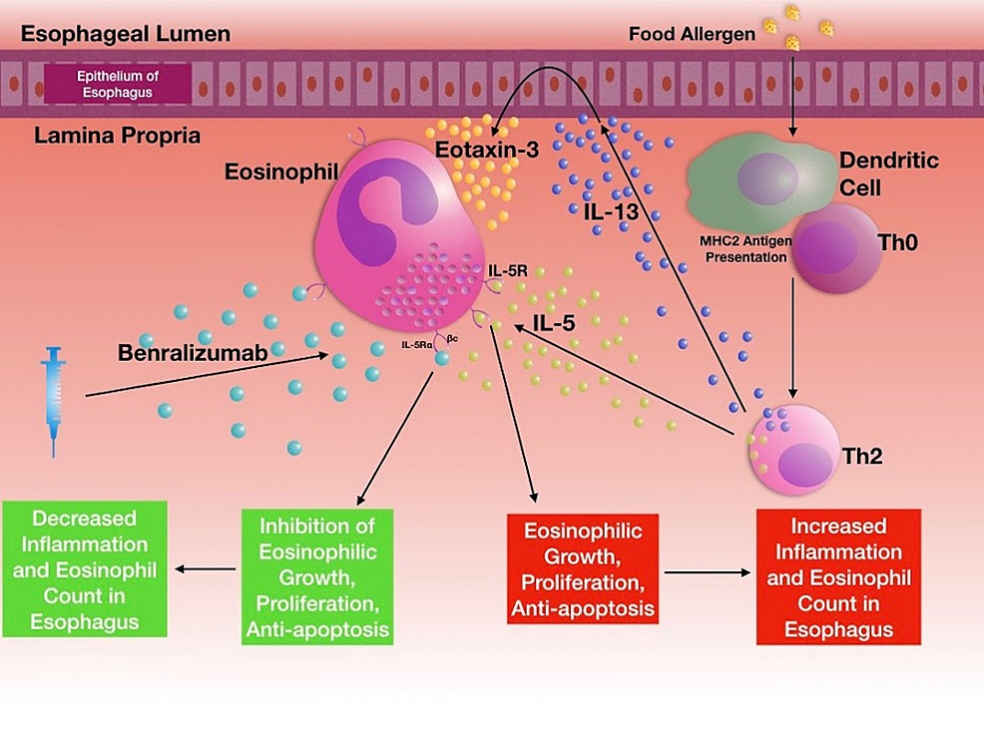
EoE pathophysiology. Dendritic cells’ activation precedes major histocompatibility complex class 2 (MHC2) antigen presentation and subsequent naive CD4+ T-cells (Th0) polarization to T-helper type-2 cells (Th2). Th2 cells then release cytokines: IL-5 (light green) and interleukin-13 (IL-13) (dark blue). IL-5 recruits eosinophils to the esophagus and stimulates eosinophilic proliferation in EoE. Benralizumab (light blue) blocks IL-5 from binding to IL-5Rα on eosinophils [10]. *MHC2*, major histocompatibility complex class 2; *Th0*, naive CD4+ T-cells; *Th2*, T-helper type-2 cell; *IL-5,* interleukin-5; *IL-13*, interleukin-13; *EoE*, eosinophilic esophagitis;* IL-5Rα*, interleukin-5-α receptor. * Note*: Original artwork created by Timothy Olsen BS, on September 17, 2020.

As a monoclonal antibody, benralizumab reduces eosinophils through two unique mechanisms: one, antagonistically blocking the alpha moiety of eosinophils' IL-5 receptors, and two, activating natural killer cells (NK-cell) for eosinophilic targeting [10]. The first mechanism competitively blocks IL-5 binding to IL-5Rα via benralizumab's Fab region, binding with a dissociation constant of 11 pM [8]. The second mechanism involves binding between benralizumab's Fc-constant region and FcɣRIII receptors on immune effector cells, such as NK-cells (dissociation constant of 45.5 nM), to cytotoxically target and destroy eosinophils (Figure 3) [8, 10]. 

While benralizumab may provide a more targeted approach than today’s standard treatments, it is nevertheless an off-label drug for EoE, costing $5,197.33/mL out-of-pocket [11]. In addition, while administering a single free sample was not enough to completely get rid of her symptoms, the sample plateaued symptoms at a lower level of discomfort compared to pre-treatment as per the patient. 

## Conclusions

As of 2021, there are no FDA-approved medications for EoE. The current standard of care includes dietary modification, PPIs, and swallowed corticosteroids. Nevertheless, these interventions are not always successful or tolerable. Reducing our patient’s EGD below 15 eos/hpf to zero eos/hpf alleviated the patient’s dysphagia, difficulty swallowing pills, and general discomfort and spontaneous nausea for one and a half months. Symptoms later returned at a lower ceiling of intensity. Future strategies may entail more precisely targeting the immunopathogenesis of EoE. This case highlights benralizumab as a potential treatment for EoE due to its specificity in repressing eosinophil activation and growth.

## References

[1] Dobbins JW, Sheahan DG, Behar J (1977). Eosinophilic gastroenteritis with esophageal involvement. Gastroenterology.

[2] (2017). Digestive diseases. Mayo Clinic, Mayo Foundation for Medical Education and Research. Mayo Clinic, Mayo Foundation for Medical Education and Research.

[3] Gensler LS (2013). Glucocorticoids: complications to anticipate and prevent. Neurohospitalist.

[4] Runge TM, Eluri S, Cotton CC, Burk CM, Woosley JT, Shaheen NJ, Dellon ES (2016). Outcomes of esophageal dilation in eosinophilic esophagitis: safety, efficacy, and persistence of the fibrostenotic phenotype. Am J Gastroenterol.

[5] American Partnership for Eosinophilic Disorders (APFED) . (2021 (2021). American Partnership for Eosinophilic Disorders (APFED). EoE. https://apfed.org/about-ead/egids/eoe/.

[6] (2021). Eosinophilic esophagitis: developing drugs for treatment guidance for industry. https://www.fda.gov/media/120089/download.

[7] Davis BP, Rothenberg ME (2016). Mechanisms of disease of eosinophilic esophagitis. Annu Rev Pathol.

[8] Federal Drug Administration. (n.d.). FASENRA (benralizumab) - US Food and Drug Administration. Accessdata.FDA.gov. https://www.accessdata.fda.gov/drugsatfda_docs/label/2017/761070s000lbl.pdf.

[9] Menzella F, Lusuardi M, Galeone C, Facciolongo N, Zucchi L (2016). The clinical profile of benralizumab in the management of severe eosinophilic asthma. Ther Adv Respir Dis.

[10] Pelaia C, Vatrella A, Bruni A, Terracciano R, Pelaia G (2018). Benralizumab in the treatment of severe asthma: design, development and potential place in therapy. Drug Des Devel Ther.

[11] Fasenra prices, coupons & patient assistance programs. https://www.drugs.com/price-guide/fasenra.

